# Overcoming challenges in ovarian visualization: a case report of a magnetic resonance imaging–guided oocyte retrieval resulting in live birth

**DOI:** 10.1016/j.xfre.2025.01.007

**Published:** 2025-01-21

**Authors:** Michael F. Neblett, Hakan Kula, David L. Walker, Katherine J. Nunemacher, David A. Woodrum, Samir N. Babayev

**Affiliations:** a Division of Reproductive Endocrinology and Infertility, Mayo Clinic, Rochester, Minnesota; b Department of Obstetrics and Gynecology, Dokuz Eylul University School of Medicine, Izmir, Turkey; c Department of Radiology, Mayo Clinic, Rochester, Minnesota; d Department of Obstetrics and Gynecology, Karabakh University, Khankendi, Azerbaijan

**Keywords:** Adhesions, case report, in vitro fertilization, magnetic resonance imaging, oocyte

## Abstract

**Objective:**

To report a successful case of magnetic resonance imaging (MRI)–guided oocyte retrieval in a patient with challenging pelvic anatomy, extensive adhesive disease, and diminished ovarian reserve, necessitated by the inability to visualize the ovaries using transvaginal or transabdominal ultrasound.

**Design:**

Case report.

**Subject:**

A 33-year-old nulligravid woman with a history of ulcerative colitis, multiple pelvic and abdominal surgeries, and significant pelvic adhesive disease.

**Exposure:**

Controlled ovarian stimulation was initiated using a gonadotropin-releasing hormone antagonist protocol, followed by sequential MRI and MRI-guided oocyte retrieval due to challenges in visualizing and accessing the ovaries.

**Main Outcome Measures:**

Successful oocyte retrieval under MRI guidance.

**Results:**

Two oocytes were retrieved from 5 aspirated follicles using MRI guidance. Both underwent intracytoplasmic sperm injection, resulting in the development of a single blastocyst, which led to a live birth via embryo transfer to a gestational carrier.

**Conclusion:**

Magnetic resonance imaging–guided oocyte retrieval represents a potential technique for patients with complex pelvic anatomy or extensive adhesive disease, where traditional ultrasound-guided approaches may be inadequate. Interdisciplinary collaboration and individualized care are crucial in optimizing outcomes for patients undergoing assisted reproduction, particularly those with challenging medical histories. Further studies comparing MRI-guided and traditional ultrasound-guided oocyte retrieval in challenging cases are warranted to determine the optimal approach for these patients.

Since its inception in the early 1980s, transvaginal ultrasound guidance for follicular aspiration has emerged as the preferred method for oocyte pickup procedures in assisted reproductive technology cycles (1, 2). The transvaginal approach is rapid and minimally invasive as the ultrasound probe comes into close proximity to the ovary. However, certain patients may still require different approaches due to challenges in locating the ovaries. Transabdominal ultrasound-guided follicular aspiration has the potential to increase the overall oocyte yield compared with the standard transvaginal method, particularly in patients with high body mass index, a history of pelvic surgeries, endometriosis, pelvic adhesions, or other challenges in visualizing the ovaries during controlled ovarian stimulation (COS) (3, 4). Other challenging cases of ovarian visualization and access may arise in patients with inflammatory bowel diseases such as ulcerative colitis, who also have heightened bowel activity, frequently associated pelvic adhesions, and a history of pelvic surgeries (5). These challenges may impede access to the ovary and increase the likelihood of complications during the procedure.

Ultrasound plays a crucial role in oocyte retrievals. Insufficient sonographic visualization presents challenges, potentially impeding the aspiration of the target material and necessitating the exploration of alternative imaging methods, such as magnetic resonance imaging (MRI) and computed tomography (CT) instead of ultrasound (6). With the advancements in imaging modalities, MRI-guided interventions have become increasingly popular, offering various applications in biopsies, drainages, and vascular interventions. Magnetic resonance imaging’s exceptional soft tissue contrast and precise anatomical visualization not only facilitate the detection of lesions inaccessible to ultrasound or CT but also reduce the risk of damage to delicate structures. Moreover, MRI-guided interventions have the added advantage of lacking ionizing radiation. Magnetic resonance imaging can image in the same location without concern about radiation dosing, which is even more heightened for women undergoing infertility treatments. Furthermore, the soft tissue resolution for MRI surpasses the other imaging modalities allowing greater ability to differentiate tissues on the basis of the imaging pulse sequences chosen (7). In this context, we present a case report of an MRI-guided oocyte retrieval necessitated by challenges in visualizing the ovaries using transabdominal and transvaginal ultrasound, performed on a patient with diminished ovarian reserve (DOR), a history of ulcerative colitis, multiple pelvic and abdominal surgeries, and significant pelvic adhesive disease.

## Case report

### Case

The patient was a 33-year-old nulligravid woman who sought a second opinion for fertility after trying to conceive with her male partner for over a year. Her medical history included ulcerative colitis, a colectomy, and a loop ileostomy 4 years prior. Her postoperative course was complicated by a fistulous tract, requiring repeat ileal pouch-anal anastomosis (J-pouch surgery).

Initially, she sought fertility evaluation at another academic center in early 2020. Laboratory results showed an antimüllerian hormone level of 0.77 ng/mL, cycle day 3 follicle-stimulating hormone level of 7.3 mIU/mL, estradiol level of 34 pg/mL, and luteinizing hormone level of 4.7 mIU/mL. Antral follicle count could not be assessed. Hysterosalpingography revealed bilateral tubal occlusion, and pelvic ultrasound showed a normal uterus but nonvisualized ovaries. Subsequent MRI indicated significant adhesive disease and limited ovarian access, making her ineligible for in vitro fertilization at that clinic.

In late 2020, she came to our clinic for a second opinion. Her gynecological history included menarche at 16 years, regular menstrual cycles, no pelvic pain, and ability to recognize ovulation. She had no history of sexually transmitted infections, and her cervical cancer screening was normal. Her partner was in good health with no history of hernia, genital surgery, trauma, or prior children. He denied tobacco, regular alcohol, or drug use. A semen analysis showed a normal total motile sperm count of 114.6 million, 65% motility, and slightly decreased morphology at 3.5% per strict Kruger criteria (8).

**Figure 1:**
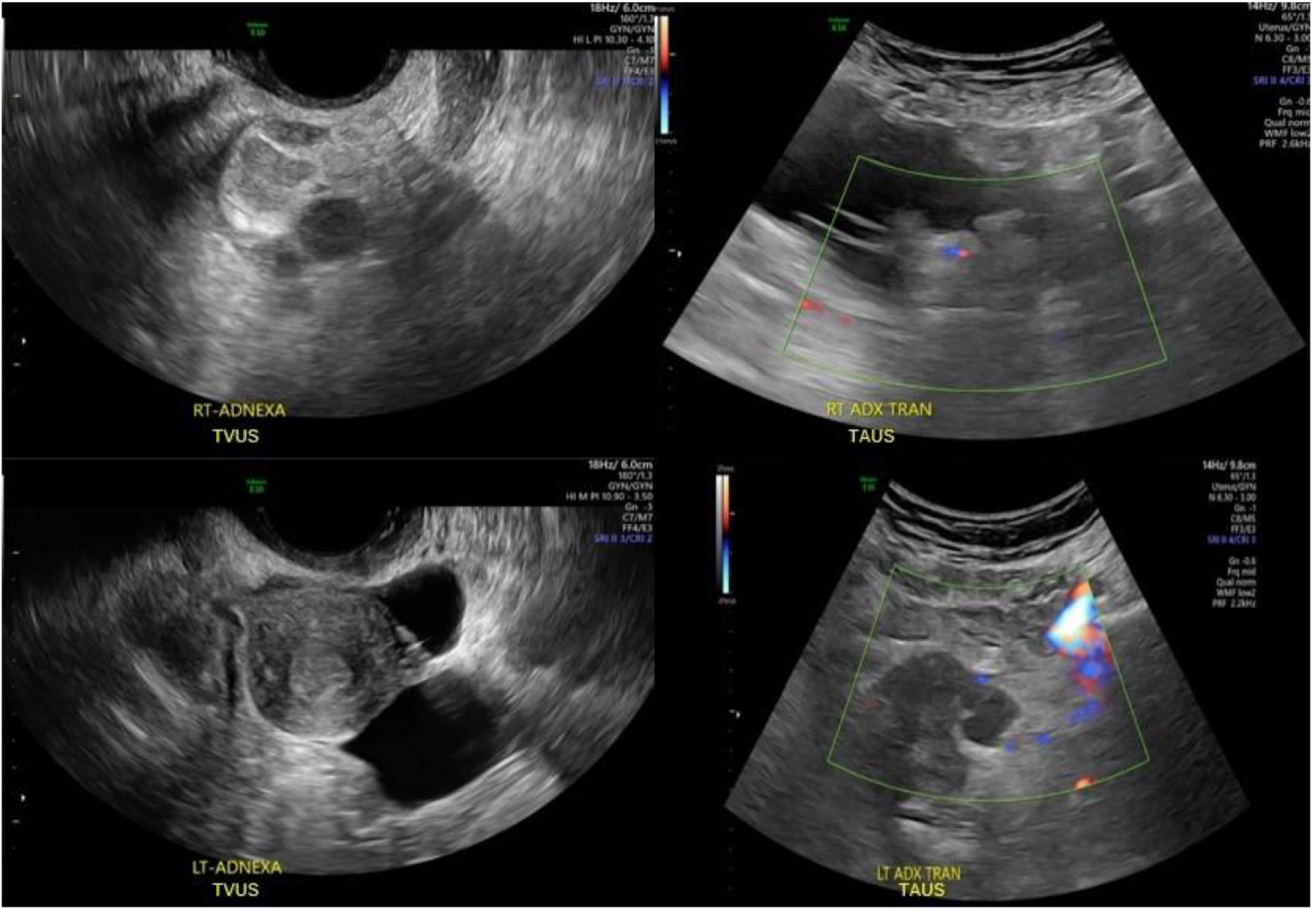
Prestimulation diagnostic ultrasound imaging. Doppler vascular imaging was employed in transabdominal ultrasound procedures and showed poor visualization of adnexal structures. ADX = adnexa; LT = left; RT = right; TAUS = transabdominal ultrasound; TVUS = transvaginal ultrasound.

Repeat ultrasound at our clinic in the late follicular phase again failed to visualize the ovaries, either transvaginally or transabdominally, and suggested a possible right hydrosalpinx along with multiple inclusion cysts bilaterally. Representative images are shown in Figure 1. After multidisciplinary consultation, we planned COS with MRI monitoring and an attempt at MRI-guided oocyte retrieval. Given her complex surgical history, the couple opted for gestational carrier treatment using autologous gametes. They completed Food and Drug Administration–required infectious disease screening. Informed consent was obtained from the patient and later her gestational carrier to write this case report.

### Stimulation/monitoring

In spring 2021, the patient began COS using a gonadotropin-releasing hormone antagonist protocol, as shown in Table 1. Baseline MRI showed severe anatomical distortion from extensive adhesions, including a large peritoneal inclusion cyst (7.3 × 13.6 × 9.4 cm) surrounding the uterus and right fallopian tube. Metal artifacts from prior surgery obscured parts of the pelvis. Both ovaries were small with few follicles, consistent with DOR. The right ovary was posterior to the inclusion cyst and medial to the external iliac vessels, whereas the left ovary was between the left external iliac artery and a pelvic small bowel loop.

**Table 1:** Medication dosing, laboratory values, and ultrasound findings throughout the stages of controlled ovarian stimulation using a gonadotropin-releasing hormone antagonist protocol.

Stimulation day	Medication dosing				Serum testing		MRI findings^a^	
	rFSH (IU)	HMG (IU)	GnRH antagonist (mcg)	hCG (IU)	E2 (pg/mL)	P4 (ng/mL)	Right ovarian follicles (mm)	Left ovarian follicles (mm)
1–3	300	150						
4	300	150			<25.0			
5	300	150						
6	300	150			42.5			
7	300	150						
8	300	150			141			
9	300	150						
10	300	150	250					
11	300	150	250		557		16	14,12
12	300	150	250					
13	300	150	250		860	0.6	17.5, 15.5, 11, 10.5	18, 11.5
14	300	150	250					
15	300	150	250		1,161	0.67	22, 20, 13.5, 13.5, 10.5	20, 12.5
16				10,000	1,460	0.81		

*Note:* Follicles were measured by averaging the diameter in 2 perpendicular planes. E2 = estradiol; GnRH = gonadotropin-releasing hormone; hCG = human chorionic gonadotropin; HMG = human menopausal gonadotropin; MRI = magnetic resonance imaging; P4 = progesterone; rFSH = recombinant follicle-stimulating hormone.

a Only follicles with a mean diameter of >10 mm included.

**Figure 2:**
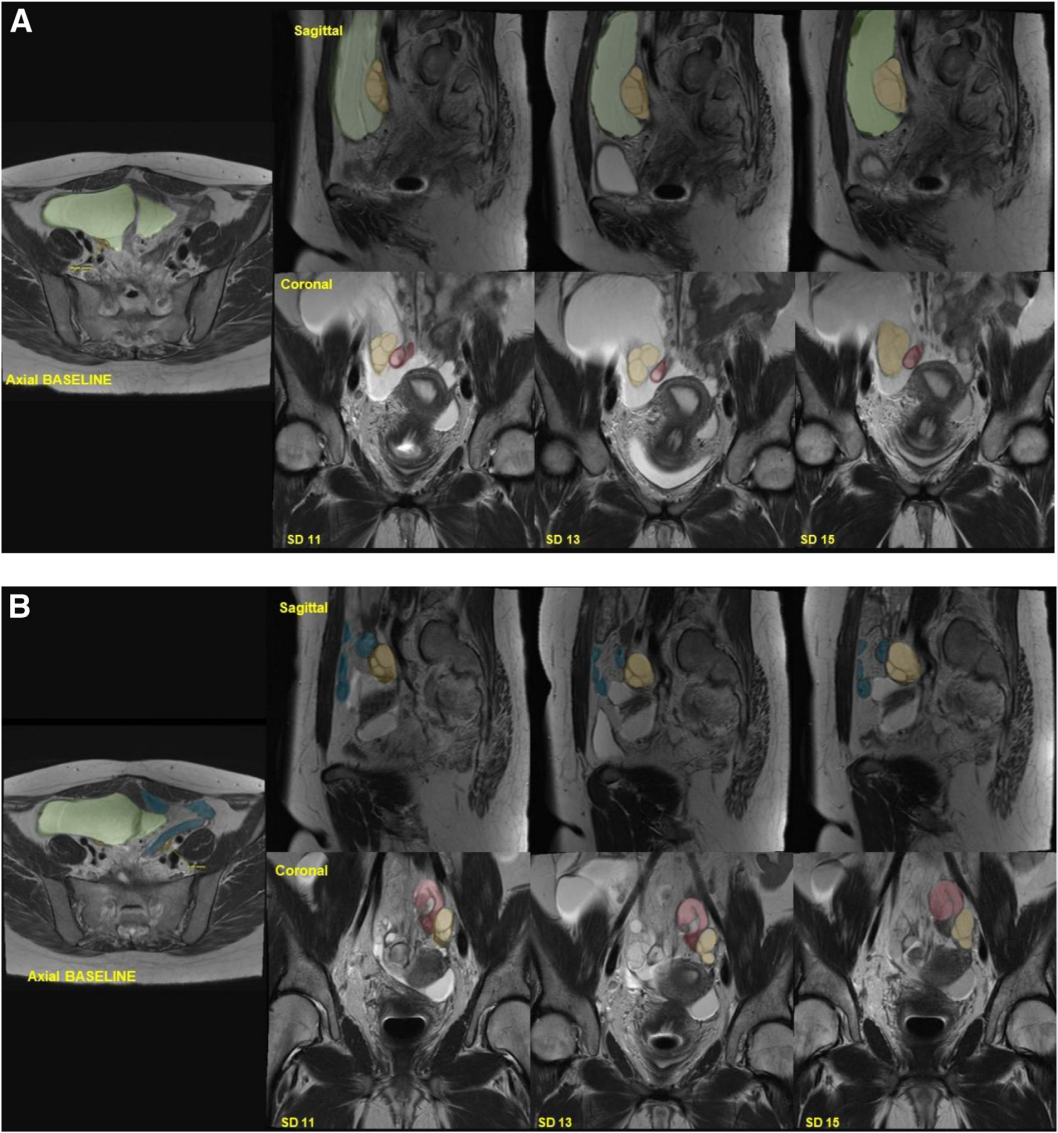
Daily monitoring and evaluation of right ovarian stimulation by using magnetic resonance imaging. (**A**) Right ovary. (**B**) Left ovary. Green shading indicates the inclusion cyst, blue shading represents the bowel segments, yellow shading denotes the ovaries, and red shading highlights the presence of a hydrosalpinx. SD = stimulation day.

Because of her anticipated poor response, she started at our clinic’s maximum doses of 300 IU of recombinant follicle-stimulating hormone and 150 IU of human menopausal gonadotropin daily. Gonadotropin-releasing hormone antagonist administration began on stimulation day 10 after a slow initial response, with projected estradiol levels between 250 and 300 pg/mL. Over 15 days, she received 4,500 IU of recombinant follicle-stimulating hormone and 2,250 IU of human menopausal gonadotropin, with 3 additional MRI monitoring sessions. Representative MRI images are shown in Figure 2. On stimulation day 13, a repeat transvaginal and transabdominal pelvic ultrasound still did not visualize the ovaries. On day 16, she received 10,000 IU of human chorionic gonadotropin to induce follicular maturation. The final MRI, a day before the human chorionic gonadotropin injection, showed leading follicles at ≥20 mm (Table 1), with most follicles expected to be >15 mm on trigger day with an assumption of the follicular growth of 2 mm per day (9). Her peak estradiol level was 1,460 pg/mL, and the progesterone level was 0.81 ng/mL on the trigger day.

### Preparation/procedure

Although MRI is generally considered a safe imaging technique, it does pose specific challenges and risks related to magnet safety (10). These risks include the potential for displacement forces and torques acting on ferromagnetic objects in the strong static magnetic field, thermal injury resulting from radiofrequency-induced heating, and the possibility of hearing damage due to the acoustic noise generated (11). Therefore, oocyte aspiration and embryology equipment were placed outside the MRI room in an adjacent control room, with minimal sterile supplies and aspiration needle near MRI. The aspiration needle was checked by our MR intervention team (MR proceduralist and MR physicist) before day of the procedure to check for safety of use and assess the degree of artifact from the aspiration needle.

**Figure 3:**
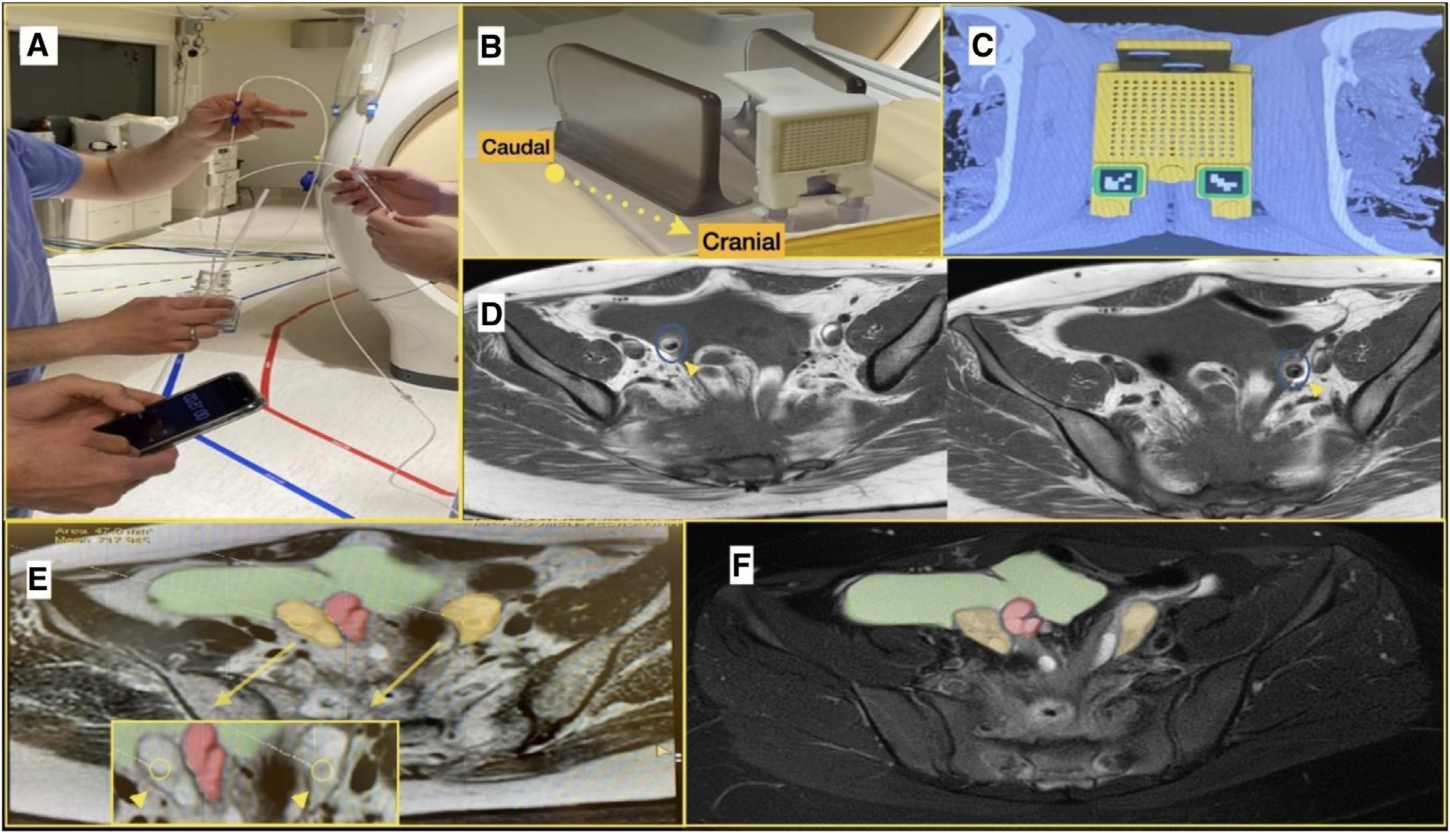
Illustration of the procedural steps of magnetic resonance imaging (MRI)–guided oocyte retrieval. (**A**) Extended tubing was attached to a 16-gauge single-lumen ovum aspiration needle (Cook), with recalibration of flow rate and vacuum pressure to optimize follicular fluid aspiration without harming oocytes. This involved aspirating normal saline to achieve a flow rate of 20–25 mL/min. (**B**) A 3 × 3 × 1–inch Acrylite sheet grid template, featuring a 4 × 6–cm array of 14-gauge-diameter holes positioned at 5-mm intervals, was placed against the perineum. (**C**) Example of a guidance grid placed over the perineum on an MR axial image. T2-weighted axial images obtained with a 3.0-Tesla MRI system were used to overlay trajectory paths, enabling precise needle placement for targeting ovarian follicles. (**D**) Placement of 2 ovum aspiration needles is shown, marked by a blue circle and yellow arrow, with visible artifacts around the needles due to their low magnetic susceptibility, similar to other MRI-guided biopsy needles. (**E**) At the initial portion of the procedure, needle placement is demonstrated with shaded colors: green for the inclusion cyst; yellow for the ovaries; and red for the hydrosalpinx. The smaller box shows a zoomed-in area highlighting ovarian follicles targeted with yellow circles and a yellow arrow. (**F**) Final imaging confirmed successful aspiration of larger measurable follicles, with no signs of hematoma. Green shading indicates the inclusion cyst, yellow shading denotes the ovaries, and red shading highlights the hydrosalpinx.

The vacuum pump (Cook Medical, Bloomington, IN) with foot pedal was kept in the control room, while extension tubing was then connected to the disposable vacuum line with a hydrophobic filter (Cook). The extended tubing was then passed into the MR interventional room via the brass penetration panel waveguide and attached to a 16-gauge single-lumen ovum aspiration needle (Cook). Due to the added resistance of extended tubing, the flow rate and vacuum pressure were recalibrated on the basis of the pump manufacturer's instructions to optimize the follicular fluid aspiration rate and avoid damage to oocytes. The flow rate was calibrated by aspirating normal saline through the aspiration needle and adjusting the vacuum pressure to achieve the recommended flow rate of 20–25 mL/min, as demonstrated in Figure 3A. Although a plastic syringe could have been used for follicular aspiration, it was not adopted due to concerns about maintaining consistent negative pressure with extended tubing in the MRI setting. Instead, the Cook aspiration system was chosen for its ability to ensure reliable flow rates, minimizing the risk of oocyte damage.

Preparations were also made for oocyte pickup outside of our in vitro fertilization laboratory. In the past, mobile laboratory carts have been effectively used for this purpose (12). However, modernized isolettes have recently been developed, offering enhanced convenience and functionality. Because of to the location of the MRI facility being different from the embryology laboratory building, we improvised by constructing a laboratory cart in the MRI control room. This cart included a dissecting microscope with heated stage, follicle tube heating block, and HEPES-buffered medium for oocyte storage and transport. Laboratory staff remained in the control room during the procedure and immediately transported the retrieved oocytes to the laboratory after completion.

The patient was first brought to the MRI anteroom for the procedure, where general anesthesia was induced via endotracheal tube intubation. She was transferred to the interventional room and positioned on the MRI table in a supine feet-first position. The lower abdomen and perineum were sterilely prepped and draped.

A transperineal approach was chosen for oocyte retrieval, similar to techniques used in MRI-guided prostate biopsies (13, 14). In a supine patient, this approach traverses the perineal skin, superficial and deep perineal fascia, perineal muscles, perineal membrane, levator ani muscles, and peritoneum until reaching the ovaries. Although the vaginal apex is anatomically closer to the ovaries used in traditional oocyte retrievals, the MRI grid’s size and rigid structure prevent its placement within the vaginal canal without compromising sterility and MRI signal integrity. Additionally, multiple attempts at transabdominal ultrasound failed to visualize the ovaries, making an abdominal approach unfeasible. The MRI-guided transperineal method was, therefore, selected as the safest and most effective option, providing optimal access and accurate visualization given the patient’s complex anatomy.

A guidance grid (Visualase Medical, Houston, TX) was placed against the perineum, and T2-weighted axial localizing images were acquired through the pelvis and grid using a 3.0 Tesla MRI system (Fig. 3B and C). The grid template consisted of a 3 × 3 × 1–inch Acrylite sheet (CRYO Industries, Rockaway, NJ, and Visualase Medical) with a 4 × 6–cm array of 14-gauge-diameter holes positioned 5 mm apart (14). Three water-filled cavities at the top of the template and in the opposite corners functioned as fusion/calibration reference markers. An integrated software application (Visualase Medical) facilitated the localization of reference marks, overlaying needle trajectories on MRI images, and calculation of insertion depths for precise needle placement. Using this guidance system, the distal end of the ovum aspiration needle was marked and guided through the grid to the designated point within the ovarian follicles. To improve efficiency, 2 needles were used, with 1 inserted into each ovary, as illustrated in Figure 3D and E.

Position confirmation and follicular fluid aspiration were performed under MRI guidance. Occasionally, minor adjustments were required to align with the target anatomy. These adjustments, similar to those in CT-guided procedures, necessitated brief interruptions to acquire repeat T2-weighted axial images, which took approximately 30–60 seconds. It is important to note that the ovum aspiration needle used in this procedure had not been specifically evaluated for MRI use in terms of safety and artifact generation. Testing demonstrated that the needle was not highly ferromagnetic, would not become a projectile with use, and did not have terrible image artifact generation. Therefore, this testing was shared with our Institutional MR Safety Committee, and they gave approval for its use. Despite the presence of some artifacts around each needle (Fig. 3D), successful aspiration was achieved under MRI guidance. Specifically, 5 follicles were aspirated from the right ovary, resulting in the retrieval of 2 oocytes. No oocytes were detected in the 3 follicles aspirated from the left ovary. The needles were removed, and the final imaging assessment revealed successful aspiration of the larger measurable follicles with no signs of hematoma, as depicted in Figure 3F. The patient was transported out of the interventional room and extubated. The procedure (approximately 3 hours) was well tolerated, and no immediate complications were observed.

### Fertilization report

Of the 2 retrieved oocytes, 1 was found to be mature and underwent intracytoplasmic sperm injection for fertilization. The decision to use this insemination method was based on the thawed sperm parameters. The sperm had been previously frozen after Food and Drug Administration testing for its potential use with a gestational carrier. The mature oocyte was successfully fertilized, developed into a blastocyst on day 5, and was vitrified. The remaining oocyte, which matured overnight, was inseminated late with intracytoplasmic sperm injection. Although fertilization occurred, the embryo was arrested at the 9-cell stage and discarded.

### Embryo transfer to gestational carrier

After sharing her story, the patient connected with a gestational carrier through social media. The carrier, a healthy 28-year-old with 2 uncomplicated vaginal deliveries, underwent a comprehensive evaluation, including a reproductive psychology consultation and infectious disease testing. After completing legal agreements, a sonohysterogram showed a normal uterine evaluation. Her endometrium was prepared using a standard medicated protocol with oral contraceptives, gonadotropin-releasing hormone agonist overlap, and estradiol. She received 6-mg oral estradiol daily and 50-mg progesterone in oil for 5 days, followed by embryo transfer on the sixth day in spring 2022. Monitoring confirmed a single intrauterine pregnancy at 7 weeks and 4 days. She continued hormone therapy until 10 weeks and had routine prenatal care. At 39 weeks and 4 days, she underwent a scheduled induction for diet-controlled gestational diabetes and delivered a healthy baby girl vaginally weighing 3,310 grams, with Apgar scores of 9 at 1 and 5 minutes.

## Discussion

To our knowledge, this is the first report documenting a successful MRI-guided oocyte retrieval resulting in a live birth. The case highlights the potential for MRI-guided oocyte retrieval in challenging clinical scenarios where transvaginal or transabdominal ultrasound-guided methods may be insufficient (15). In patients with complex medical histories, such as the one described, where pelvic adhesions, previous surgeries, and inflammatory bowel disease complicate ovarian visualization, MRI can offer superior soft tissue contrast and precise anatomical visualization (7). Furthermore, this approach may offer a considerably less invasive alternative compared with the previously used laparoscopic retrieval method before the routine adoption of vaginal approaches (16).

Performing oocyte retrieval under MRI guidance presents several technical challenges and considerations. Unlike traditional ultrasound-guided procedures, MRI-guided interventions necessitate meticulous planning and coordination because of the unique magnetic environment within the MRI suite. Time is needed for patient preparation and induction of anesthesia. Equipment, including the need for isolette or mobile carts, should be carefully considered and positioned to ensure patient safety and procedural success. This emphasizes the importance of interdisciplinary collaboration between reproductive endocrinologists, interventional radiologists, embryologists, and anesthesiologists.

Additionally, the availability and cost of MRI present notable challenges. Centers may consider bundle pricing for repeat imaging to mitigate costs. In this case, the use of a guidance grid and specialized ovum aspiration needle facilitated precise needle placement within the ovarian follicles despite the presence of artifacts around the needles under MRI. However, it is essential to acknowledge that the ovum aspiration needle used had not been specifically evaluated for MRI use and was safety tested with Institutional MR Safety Committee approval before use, highlighting the need for further research. Furthermore, because this procedure relies on MRI for guidance, it is not suitable for patients with MRI-unsafe implanted devices.

Compared with ultrasound, the loss of real-time feedback during oocyte aspiration is another limitation to be addressed, as is the inability to change trajectories and imaging planes in real time throughout the procedure. To facilitate this, multiple needle punctures may be needed, further causing more postprocedural discomfort, along with repeat scans, each taking 30–60 seconds to complete. These limitations can be challenging both in our case with patients with DOR, resulting in fewer retrieved oocytes, and in cases where patients have a high number of follicles at the time of trigger.

## Conclusion

In conclusion, MRI-guided oocyte retrieval represents a valuable technique for patients with challenging pelvic anatomy or extensive adhesive disease, where traditional ultrasound-guided approaches may be inadequate. This case underscores the importance of individualized care and interdisciplinary collaboration in optimizing outcomes for patients undergoing assisted reproduction, particularly those with complex medical histories where the alternative is the inability to provide care. Moving forward, larger-scale studies comparing the effectiveness of MRI-guided vs. traditional ultrasound-guided oocyte retrieval in challenging cases will offer valuable insights into the optimal approach for patients with complex pelvic anatomy or visualization difficulties.

## CRediT Authorship Contribution Statement

**Michael F. Neblett:** Writing – review & editing, Writing – original draft, Project administration, Methodology, Investigation, Conceptualization. **Hakan Kula:** Writing – review & editing, Writing – original draft, Visualization. **David L. Walker:** Writing – review & editing, Project administration, Methodology, Investigation, Conceptualization. **Katherine J. Nunemacher:** Writing – review & editing, Project administration, Methodology, Investigation, Conceptualization. **David A. Woodrum:** Writing – review & editing, Supervision, Software, Resources, Project administration, Methodology, Investigation, Conceptualization. **Samir N. Babayev:** Writing – review & editing, Writing – original draft, Supervision, Resources, Project administration, Methodology, Investigation, Conceptualization.

## Declaration of Interests

M.F.N. has nothing to disclose. H.K. has nothing to disclose. D.L.W. has nothing to disclose. K.J.N. has nothing to disclose. D.A.W. reports consulting fees for Boston Scientific on educational issues on magnetic resonance–guided cryoablation outside the submitted work. S.N.B. reports ownership interest in McKesson, and honoraria on educational issues from Medscape.
